# Resolving the taxonomic conundrum in *Graphoderus* of the east Palearctic with a key to all species (Coleoptera, Dytiscidae)

**DOI:** 10.3897/zookeys.574.7002

**Published:** 2016-03-28

**Authors:** Sandra Holmgren, Robert Angus, Fenglong Jia, Zhen-ning Chen, Johannes Bergsten

**Affiliations:** 1 Department of Zoology, Swedish Museum of Natural History, Box 50007, SE-104 05 Stockholm, Sweden; 2 Department of Zoology, Stockholm University, SE-106 91 Stockholm, Sweden; 3 Department of Life Sciences (Insects), The Natural History Museum, Cromwell Road, London SW7 5BD, UK; 4 Museum of Biology, Sun Yat-sen University, Guangzhou, China; 5 Biology and Geography School, Qinghai Normal University, Wusi West Road 38#, 810000, Xining, Qinghai Province, China

**Keywords:** *Graphoderus*, east Palearctic, Nearctic, *Graphoderus zonatus*, *Graphoderus perplexus*, *Graphoderus elatus*, male genitalia, Yenisei-Angara river, dimorphic females

## Abstract

The Holarctic diving beetle genus *Graphoderus* (Dytiscinae, Aciliini) contains relatively few and well-known species but these may still be difficult to identify based on external characters. A taxonomic problem in the eastern Palearctic was discovered that relates to the Palearctic *Graphoderus zonatus* (Hoppe, 1795) and the Nearctic *Graphoderus perplexus* Sharp, 1882. Based on qualitative and quantitative characters, especially on male genitalia which have been poorly studied in the past, it is shown that eastern Palearctic specimens identified by previous authors as either of the two species in fact belongs to a third species. The synonymized name *Graphoderus elatus* Sharp, 1882, is reinstated as a valid species (**stat. n.**) and a lectotype is designated from the mixed syntype series. The male genitalia of all known *Graphoderus* species have been examined and an illustrated identification key to the genus is provided. The three species in the complex of focus, *Graphoderus elatus*, *Graphoderus zonatus* and *Graphoderus perplexus* are found to have allopatric distributions; *Graphoderus perplexus* in the Nearctic region, *Graphoderus zonatus* in the west Palearctic region and eastwards to the Yenisei-Angara river and *Graphoderus elatus* east of the Yenisei-Angara river. All previous records of either *Graphoderus zonatus* or *Graphoderus perplexus* in the east Palearctic, east of the Yenisei-Angara river turned out to be misidentified *Graphoderus elatus*. This conclusion also brings with it that dimorphic females, thought only to be present in the single subspecies *Graphoderus zonatus verrucifer* (CR Sahlberg, 1824), proved to be present also in a second species, *Graphoderus elatus*. The dimorphic female forms is either with dorsally smooth elytra and pronotum or conspicuously granulated elytra and wrinkly pronotum. As has been shown in *Graphoderus zonatus verrucifer* there is a correlation between the occurrence of granulate female forms in a population and an increase in the number of adhesive discs on pro- and mesotarsus in males within *Graphoderus elatus*.

## Introduction

The genus *Graphoderus* Dejean, 1833 consists of medium sized (10–16 mm) diving beetles of the family Dytiscidae. Adults are dorsally testaceous to rufous with black irrorations and except for one species they all have two transverse black bands across the pronotum (Nilsson and Holmen 1995, Larson et al. 2000). Both adults and larvae are found in ponds, smaller lakes, bogs or wetlands (Nilsson and Holmen 1995, Larson et al. 2000). Larvae feed mainly on planktonic microcrustaceans but also on larvae and pupae of mosquitoes (Culicidae) (Galewski 1975, 1990). Adults are predatory on crustaceans, insects and worms (Galewski 1990). Oviposition, and larval and pupal development takes place from April or May to October, and in some years there is also a partial second generation (Galewski 1990, Hilsenhoff 1993, Nilsson and Holmen 1995, Foster 1996). Adults overwinter in aquatic habitats, under *Sphagnum* in bogs or hidden in bottom sediments of water-bodies which do not dry out (Galewski 1990, Hilsenhoff 1993). Males have enlarged protarsomeres I–III forming a palette with adhesive discs and mesotarsomeres I–III may be with or without adhesive discs (Nilsson and Holmen 1995).

### Current diversity and state of affairs

Currently, *Graphoderus* is regarded as consisting of eleven species and two subspecies, all distributed in the Holarctic realm (Nilsson 2001, 2015). The Nearctic species of *Graphoderus* were recently treated by Larson et al. (2000) who listed five species: *Graphoderus liberus* (Say, 1825), *Graphoderus perplexus* Sharp, 1882, *Graphoderus occidentalis* Horn, 1883, *Graphoderus fascicollis* (Harris, 1828) and *Graphoderus manitobensis* Wallis, 1933. The first three are transcontinental whereas the latter two are more restricted in distribution to east-central North America (Larson et al. 2000). Nilsson and Holmen (1995) treated the four west Palearctic species: *Graphoderus austriacus* (Sturm, 1834), *Graphoderus cinereus* (Linnaeus, 1758), *Graphoderus bilineatus* (De Geer, 1774) and *Graphoderus zonatus* (Hoppe, 1795) with the latter divided into the subspecies *Graphoderus zonatus zonatus* (Hoppe, 1795) and *Graphoderus zonatus verrucifer* (CR Sahlberg, 1824), following Nilsson (1986). How far the distribution of these species extends into the east Palearctic is somewhat uncertain, but all reach at least west Siberia (Nilsson 2003a, Nilsson and Hájek 2015). *Graphoderus austriacus* and *Graphoderus zonatus* are regarded as extending further into Far East Russia where they meet the two exclusively east Palearctic species: *Graphoderus adamsii* (Clark, 1864) and *Graphoderus bieneri* Zimmermann, 1921 (Lafer 1989).

This order was shaken when Nilsson et al. (1999) reported the Nearctic species *Graphoderus perplexus* to also occur in the east Palearctic and we started to discover additional *Graphoderus perplexus*-like specimens from the east Palearctic.

### *Graphoderus* species in the spotlight

One of the species, *Graphoderus bilineatus*, has received increased attention after it was put on the EU list of species in Annex II under the Habitats Directive. EU member states were required to designate special areas of conservation for the species in Annex II and report on their conservation status. Environmental agencies in several EU countries have since made focused inventories of this species to get better data on its occurrence, distribution and abundance (Hájek 2004, Hendrich and Balke 2005, Kalniņš 2006, Hendrich and Spitzenberg 2006, Sierdsema and Cuppen 2006, Cuppen et al. 2006, Naturvårdsverket 2011, Hendrich et al. 2011, Iversen et al. 2013, Przewoźny et al. 2014, Scheers 2015). Results of the inventories have also contributed to new studies investigating the distribution of *Graphoderus bilineatus* in neighboring countries (Kálmán et al. 2011, Csabai et al. 2015).

Another *Graphoderus* species which has received substantial attention lately is *Graphoderus zonatus* and in particular its subspecies *Graphoderus zonatus verrucifer*, as its females are dimorphic with one morph dorsally smooth like the male and the other morph with a peculiar wrinkly pronotum and roughly granulated elytra (Nilsson 1986, Nilsson and Holmen 1995, Bergsten et al. 2001, Härdling and Bergsten 2006, Karlsson-Green et al. 2013, 2014). The two morphs co-occur in varying proportions in different populations and it was shown by Bergsten et al. (2001) that the proportion of the granulate morphs in a population was significantly correlated with suction cup characteristics of the male’s pro- and mesotarsal palettes. With a higher proportion of the granulated female morph male pro- and mesotarsal palettes got wider, the three large suction cups got larger and the smaller suction cups got smaller and more numerous (Bergsten et al. 2001). The female granulate morph was interpreted as an antagonistic character evolved in an arms race with male suction cups over aspects in the mating, e.g. frequency and timing (Arnqvist and Rowe 2005). The antagonistic nature of the dorsal female sculpture to the function of the mechanically working male suction cups was inferred mathematically from first principles by Bergsten and Miller (2007) and later shown empirically (Karlsson-Green et al. 2013). Both theoretical (Härdling and Bergsten 2006) and empirical (Karlsson-Green et al. 2014) work has been conducted to try to understand the role of selection and drift for the distinct morphs to be able to co-occur over time.

### The taxonomic conundrum in the east Palearctic

In 1882, Sharp (1882) described a *Graphoderus* species with Holarctic distribution, *Graphoderus elatus* Sharp, 1882, based on material from both Canada and east Siberia. Sharp (1882) separated this species from the Nearctic species *Graphoderus perplexus*, described in the same work, based on the number of adhesive discs on pro- and mesotarsus. Both species were distinguished from the Palearctic species *Graphoderus zonatus* by being narrower in front and having wider epipleura. Horn (1883) was not convinced that the male tarsal characters given by Sharp (1882) indicated separate species and made *Graphoderus fascicollis*, *Graphoderus perplexus* and *Graphoderus elatus* synonyms of the Palearctic *Graphoderus cinereus*. Zimmermann (1920) disagreed with Horn (1883) and instead placed *Graphoderus perplexus* and *Graphoderus elatus* as synonyms of *Graphoderus zonatus*. We will refer to this “species group” (*Graphoderus zonatus*, *Graphoderus perplexus* and *Graphoderus elatus*) which might not be closely related, as the *zonatus*-species complex and it is distinguished from other *Graphoderus* species in that the black pronotal bands mostly neither reach the anterior nor the posterior pronotal margins (Nilsson and Holmen 1995, Larson et al. 2000). Further, Gschwendtner (1937) mentioned that *Graphoderus perplexus* and *Graphoderus elatus* were not particularly different from *Graphoderus zonatus*. Instead Wallis (1939) was the one who separated *Graphoderus perplexus* and *Graphoderus elatus* from *Graphoderus zonatus* by the same characters which Sharp (1882) used – wider epipleura and a body shape more narrowed in front. Also, Wallis (1939) specified differences between *Graphoderus zonatus* and *Graphoderus perplexus*/*Graphoderus elatus* in the bifurcation of the chitinous ring enclosing the genitalia. However, after having examined over 100 males, he proposed that *Graphoderus perplexus* and *Graphoderus elatus* could not be separated from each other based on the number of adhesive discs and he synonymized them as the same species with *perplexus* as the valid name. Zaitsev (1953) (English translation (Zaitsev 1972)) described the Russian fauna of Dytiscidae in 1953 and did not mention *Graphoderus perplexus* as occurring in the Palearctic region. However Zaitsev (1972) put Sharp’s *Graphoderus elatus* type from east Siberia as a synonym of *Graphoderus zonatus* and also reported *Graphoderus zonatus* to be distributed in North America. Later, in the treatment of the Russian Far East Dytiscidae by Lafer (1989), the *Graphoderus elatus* type from east Siberia was not referred to at all, instead Lafer only mentioned *Graphoderus zonatus* from the *zonatus*-species complex to occur in the Palearctic. Yet, in 1999 Nilsson et al. (1999) studied newly collected material from Kamchatka (Russian Far East) and identified the material of a *Graphoderus* species as *Graphoderus perplexus* based on the number of tarsal adhesive discs which was significantly fewer than the material from Urup, Kuril Islands, instead identified as *Graphoderus zonatus* (Nilsson et al. 1997). This resurrected the old hypothesis by Sharp (1882) of a *Graphoderus* species occurring in both the Nearctic and east Palearctic realms, now under the name *Graphoderus perplexus*. This was also transferred to the world catalogue (Nilsson 2001, 2015) and the Palearctic catalogue (Nilsson 2003a, Nilsson and Hájek 2015). There is however a problem with using the number of tarsal suction cups as a sole distinguishing character because of its variation and correlation with female counter-adaptions (Bergsten et al. 2001). The number of suction cups was used by Nilsson (1986) to delimit the two subspecies *Graphoderus zonatus zonatus* and *Graphoderus zonatus verrucifer* but only based on Swedish material. In Sweden the two subspecies could be delimited geographically in that south of a diagonal line across southern Sweden no granulate females were known (Nilsson 1986). On average the number of both pro- and mesotarsal suction cups were higher in the northern subspecies (*Graphoderus zonatus verrucifer*) but the variation overlapped (Nilsson 1986). Populations of *Graphoderus zonatus verrucifer* occur as well in Italy but these populations were earlier considered to be a variety of *Graphoderus cinereus* (var. *bertolinii* Seidlitz, 1887), which Pederzani (1986) instead reported to belong to a relict population of *Graphoderus verrucifer* (CR Sahlberg, 1824), new to Italy. The same year Nilsson (1986) changed the status of *Graphoderus verrucifer* to a subspecies of *Graphoderus zonatus*. Yet, how to delimit the subspecies of *Graphoderus zonatus* in the eastward extension of the distribution across Russia and Siberia to Japan is poorly understood (Nilsson and Holmen 1995). Both subspecies of *Graphoderus zonatus* are listed for Mongolia and all records of *Graphoderus zonatus* from China and Japan are considered to be *Graphoderus zonatus zonatus* whereas all western and eastern Siberian as well as Far East Russian *Graphoderus zonatus* are considered to be *Graphoderus zonatus verrucifer* (Nilsson 2003a, Nilsson and Hájek 2015). The number of tarsal suction cups in the Kamchatka material is in fact partly within the documented range for *Graphoderus zonatus zonatus* (Nilsson 1986, Nilsson et al. 1999) which was probably excluded only on the basis of a presumed more southern distribution.

In summary then, there is an unresolved taxonomic conundrum in the east Palearctic. Is there really a partly Holarctic *Graphoderus* species spanning both sides of Beringia? Are there other distinguishing characters apart from the number of males’ suction cups to shed light on the *zonatus*-species complex? We are especially interested in evaluating the diagnostic power of the male genitalia because the genitalia in *Graphoderus* have not been as extensively used as in e.g. Agabini due to their partly soft-tissue nature (Nilsson and Holmen 1995). There is no reason to believe that it is less informative than the genitalic characters in the sister-group *Acilius* Leach, 1817 (Bukontaite et al. 2014) which recently helped to solve a taxonomic confusion in the Nearctic region (Bergsten and Miller 2006). If there is no Holarctic *Graphoderus* how should we treat *Graphoderus elatus* based on the syntype series including both continents? In the updated world catalogue (Nilsson 2015), *Graphoderus elatus* is listed with a “?” as a synonym under *Graphoderus perplexus* and in the updated Palearctic catalogue (Nilsson and Hájek 2015) *Graphoderus perplexus* is listed as occurring in far east Russia and Nearctic but without the synonym *Graphoderus elatus*.

The aim of this study is to 1) resolve the taxonomic conundrum of the *zonatus*-species complex in the east Palearctic, 2) evaluate the usefulness of the male genitalia for species identification and delimitation in *Graphoderus* and 3) to provide an identification key and iconography with habitus and male genitalia images of all *Graphoderus* species.

## Methods

Examined material came from the following collections referred to by their abbreviation:

BMNH
 The Natural History Museum, London, United Kingdom 


CNC
 Canadian National Collection of Insects, Ottawa, Ontario, Canada 


IRCW
University of Wisconsin (Ex. Coll W. Hilsenhoff), Madison, Wisconsin, USA 


NHRS
Swedish Museum of Natural History, Stockholm, Sweden 


OMNH
Osaka Museum of Natural History, Osaka, Japan 


SYSU
 Biological Museum of Sun Yat-sen University, Guangzhou, China 


ZIN
Zoological Institute, Russian Academy of Sciences, St Petersburg, Russia 


ZMUM
Zoological Museum, Moscow State University, Moscow, Russia 


### Measurements

**Figure 1.**
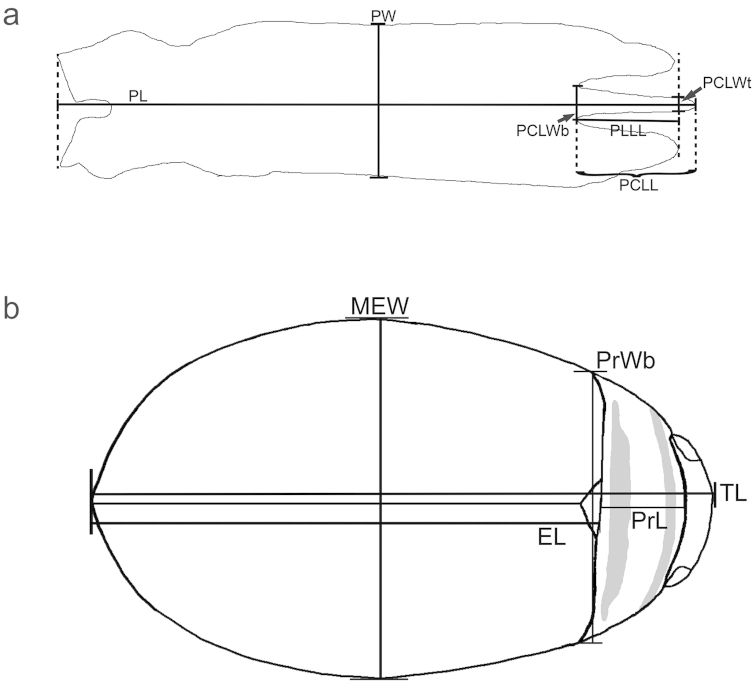
Explanations of measurements for penis (**a**) and body (**b**). PW = penis width, PL = penis length, PCLL = penis central lobe length, PLLL = penis lateral lobe length, PCLWb = penis central lobe width at base and PCLWt = penis central lobe width at level of lateral lobe apex, TL = total body length, EL = elytral length, MEW = maximum elytral width, PrL = pronotal length and PrWb = pronotal width at base.

Genitalia were prepared from 57 male specimens of Nearctic *Graphoderus perplexus* and from sixteen males of the *Graphoderus perplexus*-like specimens from east Palearctic, shown in this paper to be *Graphoderus elatus* Sharp, 1882. Six measurements were then taken (in micrometers, µm) from photographs of the genitalia, with focus at the anterior lobes of the penis. The camera was an infinity X, mounted on an Olympus SZX12 stereomicroscope using the program DeltaPix InSight v4.0.9. The measurements were; PW = penis width (at midway between apex and base), PL = penis length, PCLL = penis central lobe length, PLLL = penis lateral lobe length, PCLWb = penis central lobe width at base and PCLWt = penis central lobe width at level of lateral lobe apex (Fig. 1a). Another five measurements were also taken from seventeen males and ten females of *Graphoderus elatus* from the east Palearctic; TL = total body length, EL = elytral length, MEW = maximum elytral width, PrL = pronotal length and PrWb = pronotal width at base (Fig. 1b). Total body length (TL) was also measured for the 57 Nearctic male specimens of *Graphoderus perplexus*.

Four ratios, PL/TL; PW/PL; PLLL/PCLL and PCLWt/PCLWb, were calculated from the measurements to test the hypothesis that there were no differences in ratios between the populations from the two continents. The ratios were tested with separate, independent 2-group Mann-Whitney tests with default settings in R version 3.2.2 2015-08-14 (R Team Core 2015). Bonferroni correction, a multiple-test correction, was used to compensate for testing several measurements from the same individuals against each other (Dunn 1961). Boxplots of the ratios with median and ± 25% of the ratio values (whiskers showing minimum and maximum ratios), were also made in R.

The number of adhesive discs on pro- and mesotarsus was counted for seventeen male specimens of Nearctic *Graphoderus perplexus* and fourteen males of *Graphoderus elatus* from the east Palearctic, excluding males from populations with or possibly with granulated females. The average number of adhesive discs from left and right pro- and mesotarsus of each specimen was calculated and the numbers were tested against the hypothesis that there were no differences between the populations from the two continents. This was tested with independent 2-group Mann-Whitney tests with default settings in R, in which also boxplots were made. The tests were repeated, this time including males from populations with granulated females which did not alter the results (not shown).

### Images

Habitus photographs of the species in dorsal view were taken with a Canon EOS 5D DSLR, and a Canon 100mm 2.8L Macro lens mounted on a motorized rail (Stackshot) from Cognisys. The photos were stacked in Zerene Stacker v1.04 and edited in Digital Photo Professional v3.13.20 and Adobe Photoshop CS5. Photographs of the male genitalia in dorsal and lateral view (slightly dorsolateral to avoid penis apex being hidden by tips of the parameres) were taken with the same infinity X camera and Olympus SZX12 stereomicroscope as above. The photographs shown in Figure 7, 10 and 11 were taken with a Leica M125 stereomicroscope + Canon EOS 550D digital camera in the Sackler Bioimaging Laboratory of the Natural History Museum, London. They were stacked using Helicon Focus software. The photographs of the genitalia in dorsal view were edited in Adobe Photoshop CS5 and the photographs of the lateral view were used as aid to make line drawings of each species penis in Adobe Illustrator CS5. The final plates were made in Adobe Photoshop CS5.

## Results

**Figure 2.**
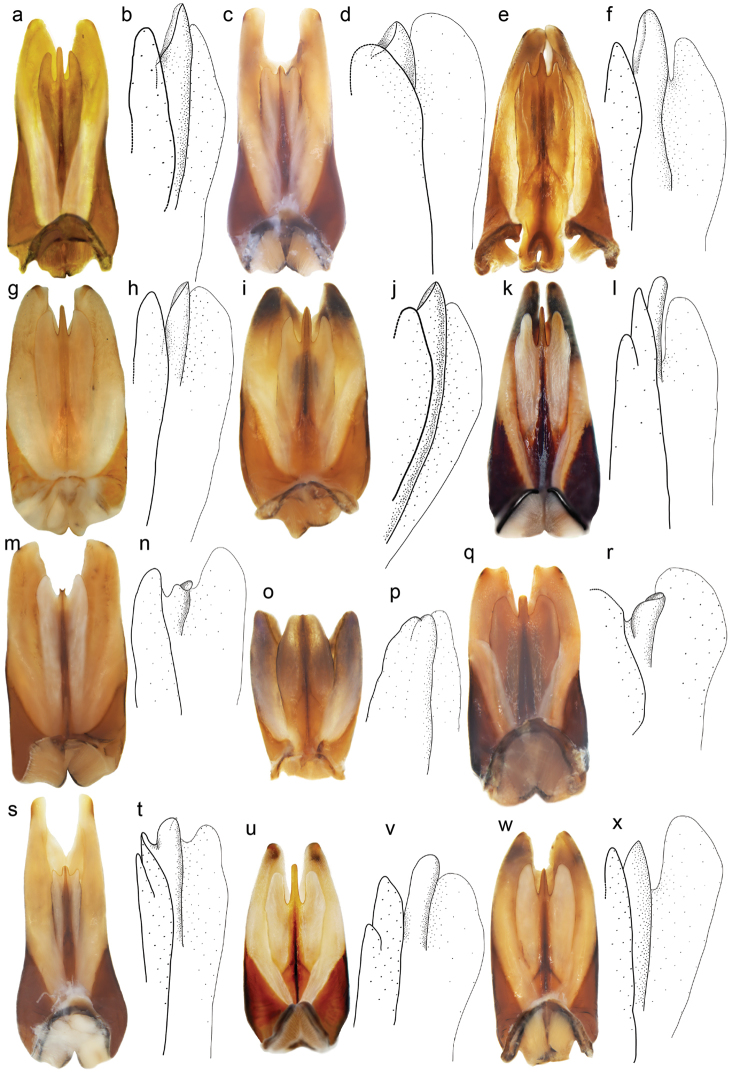
Genitalia in dorsal view (photo), with detailed lateral view of penis apex (line drawing) of all *Graphoderus* species. **a–b** *Graphoderus adamsii* **c–d** *Graphoderus austriacus* **e–f** *Graphoderus bieneri* **g–h** *Graphoderus bilineatus* **i–j** *Graphoderus cinereus* **k–l** *Graphoderus elatus* [dorsal view, processed by E. Binkiewicz] **m–n** *Graphoderus fascicollis* **o–p** *Graphoderus liberus* **q–r** *Graphoderus manitobensis* **s–t** *Graphoderus occidentalis* **u–v** *Graphoderus perplexus* [dorsal view, processed by E. Binkiewicz] **w–x** *Graphoderus zonatus*.

Examination of male genitalia in the *zonatus*-species complex revealed that the lateral view of the central lobe at the penis’ trifid apex was highly diagnostic to separate *Graphoderus zonatus* from *Graphoderus perplexus* and *Graphoderus elatus* (Fig. 2). In *Graphoderus zonatus*, in lateral view the dorsal margin of the central penis lobe forms an even convex curve (Fig. 2x). In *Graphoderus perplexus* and *Graphoderus elatus* the same view shows a concave shape (Fig. 2v, l). The shape was identical in both subspecies of *Graphoderus zonatus*, including material from the population in the Italian Alps, monomorphic for the granulated female morph. Examination of east Palearctic material based on this character showed that all material east of the Yenisei river and its headwater tributary Angara river was not *Graphoderus zonatus* although it had often been misidentified as *Graphoderus zonatus* (Zaitsev 1972, Lafer 1989, Mori and Kitayama 1993, Nilsson 1995, Nilsson et al. 1999). As well, all records from the northernmost peninsula on Hokkaido in Japan of *Graphoderus zonatus* are based on misidentifications (Mori and Kitayama 1993). The easternmost record of *Graphoderus zonatus* we have studied is a male taken by RB Angus in 1970 at Dachnaya, just west of Irkutsk Lat. 52.1220°N Lon. 104.0840°E.

Reported *Graphoderus zonatus* from Sakhalin and Kuril Islands (Nilsson et al. 1997, 1999) are based on misidentifications as shown by reexamination of the material in Nilsson’s collection donated to NHRS in 2013. Records of *Graphoderus zonatus* from northeast China are with all certainty also based on misidentifications (Nilsson 1995), as we found material from Inner Mongolia, Heilongjiang and Qinghai to have the concave shape of the central penis lobe. This shape was also identified in beetles from northeast Mongolia, Onon river, but the distribution of *Graphoderus zonatus* in the north-central part of Mongolia (Shaverdo et al. 2008) is more uncertain. Apart from the male genitalia, *Graphoderus zonatus* can also be distinguished from *Graphoderus perplexus* and *Graphoderus elatus* by having narrower epipleura posteriorly.

### Granulate females and adhesive discs

**Figure 3.**
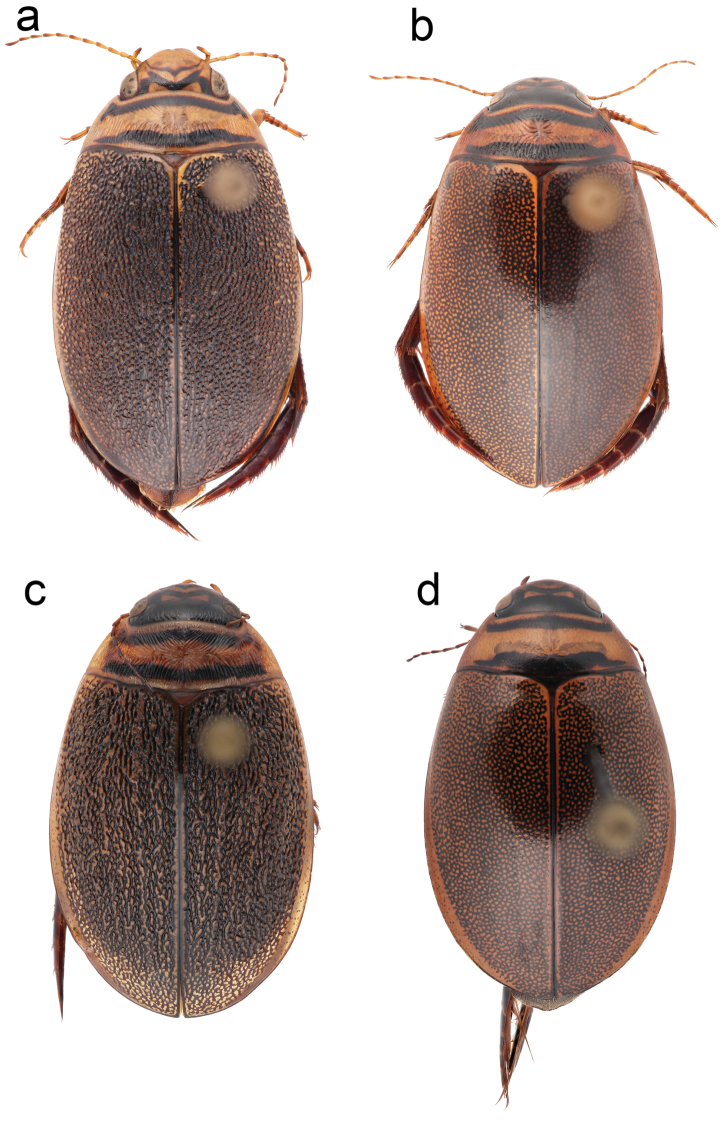
Female dimorphism in *Graphoderus zonatus* (**a–b**) and *Graphoderus elatus* (**c–d**). **a, c** granulated female elytra **b, d** smooth female elytra.

The realization that true *Graphoderus zonatus* could be distinguished by the shape of male genitalia in lateral view and based on this also could be inferred not to occur east of Yenisei-Angara river brought about an enticing novelty. *Graphoderus zonatus* was no longer the only *Graphoderus* species with dimorphic females, one morph of which had elytra granulated and pronotum wrinkled and the other morph which had smooth elytra like the males. Granulated females have been reported east of the Yenisei-Angara river, e.g. from the Kuril Island Urup, which has been seen as evidence for the subspecies *Graphoderus zonatus verrucifer* (Nilsson et al. 1997). But our examinations of the same Kuril material showed that the males from that population were not *Graphoderus zonatus*. We have also found granulated females from Inner Mongolia (leg. Li, Chunyuan and Chaoqun) and Yakutsk with a male from the latter population rejecting the identity as *Graphoderus zonatus*. As well, in the material from North Sakhalin we found females with smooth elytra, however the male from the same population had a higher number of suction cups indicating that female dimorphism can occur within the same population. Closer comparison of the granulated females showed a somewhat more irregular granulated structure on the elytra, more elongated convexities of the granules and a stronger tendency towards forming longitudinal lines in the east Palearctic material (Fig. 3) but this difference is so far based on too few granulate females to be considered conclusive. Just as predicted by the arms race hypothesis males from granulated populations showed a higher number of adhesive discs on the tarsi. Males from the North Sakhalin and Urup populations had 59-66 protarsal suction cups and 29-31 mesotarsal suction cups. This should be compared with 28-47 protarsal and 14-20 mesotarsal suction cups from localities without granulated females. Granulated *Graphoderus* females have never been found in the Nearctic region but females of the Nearctic species may sometimes have wrinkles on the pronotum.

### Quantitative morphometrics

**Figure 4.**
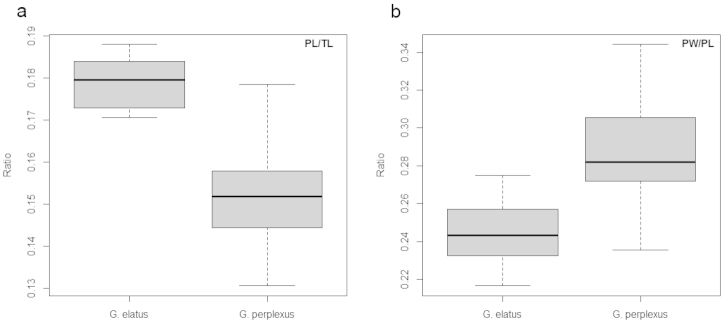
Boxplots with the variation in PL/TL (**a**) and PW/PL (**b**). Ratios for *Graphoderus elatus* and *Graphoderus perplexus* in penis length over total body length (PL/TL) and penis width over penis length (PW/PL), the box represents median ± 25% and whiskers show minimum and maximum values.

**Figure 5.**
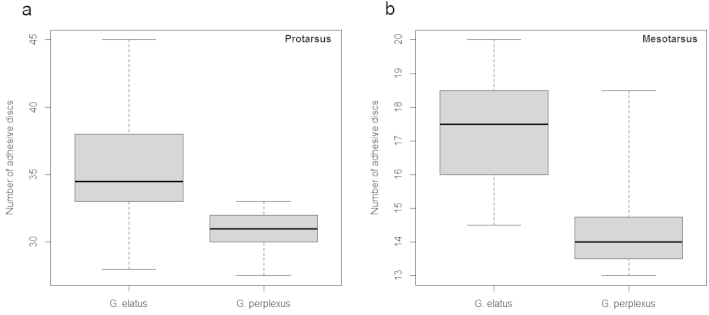
Boxplots with the variation in number of adhesive pro- (**a**) and mesotarsal (**b**) discs. Number of adhesive discs for *Graphoderus elatus* and *Graphoderus perplexus*, the box represents median ± 25% and whiskers show minimum and maximum numbers.

**Figure 6.**
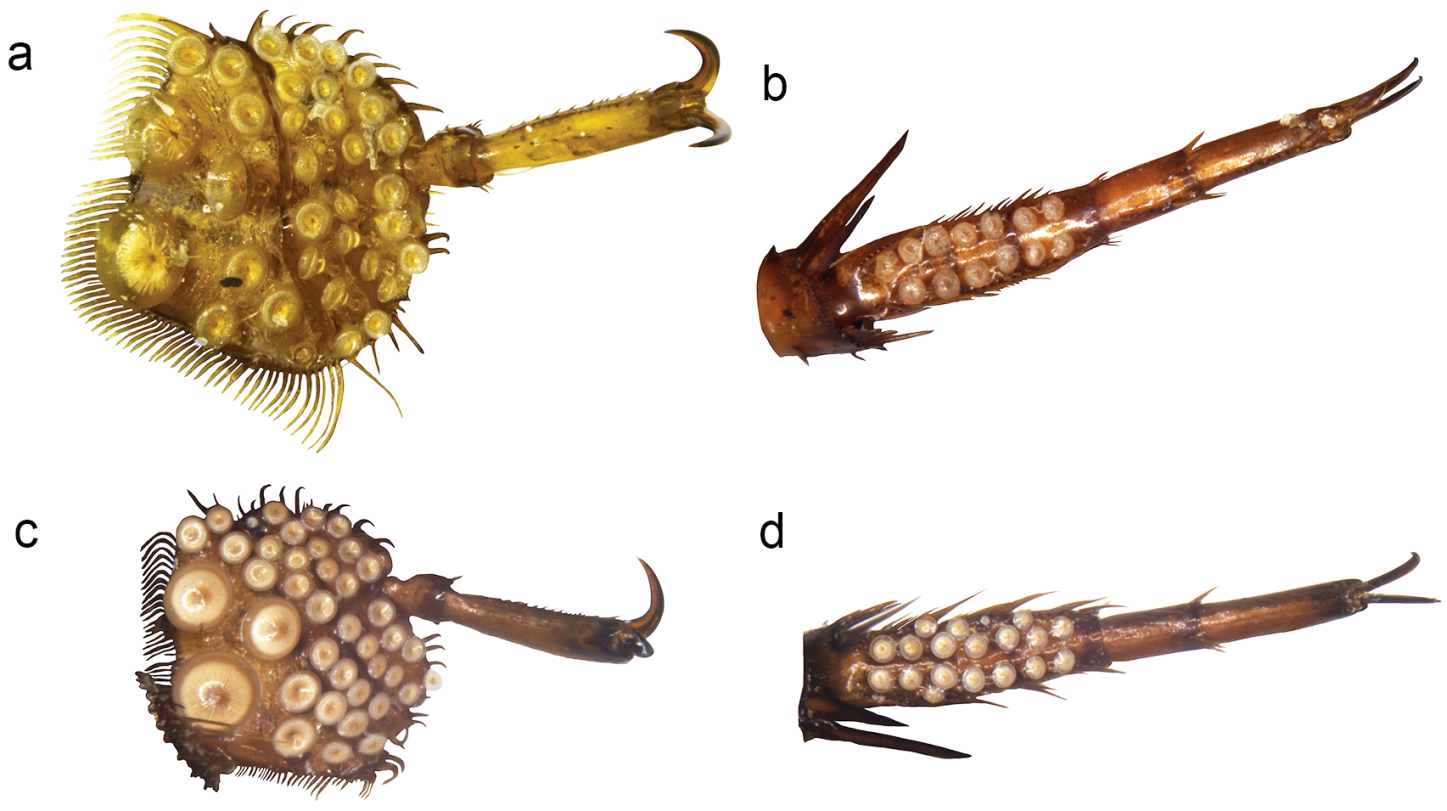
Adhesive discs on pro- (**a, c**) and mesotarsus (**b, d**). **a–b** *Graphoderus perplexus* **c–d** *Graphoderus elatus*.

As Nearctic *Graphoderus perplexus* and east Palearctic *Graphoderus elatus* have a similar concave shape of the male central penis lobe in lateral view, various characteristics of the male genitalia were quantified to test for con- or heterospecificity (see Methods). In particular we had from initial examination noted that the penis, as well as the entire genitalic package with parameres, seemed to be notably longer in the east Palearctic specimens (compare Fig. 2k with 2u). Two separate Mann-Whitney tests showed that *Graphoderus elatus* from the east Palearctic are distinguished from Nearctic *Graphoderus perplexus* in the male genitalia by significant difference in the ratio PL/TL (relative penis length, P < 0.001) (Fig. 4a) and in PW/PL (penis shape, P < 0.001) (Fig. 4b) (Table 1). Two separate Mann-Whitney tests also showed that Nearctic *Graphoderus perplexus* had significantly fewer adhesive discs on both pro- and mesotarsus (P < 0.001) (Fig. 5). Figure 6 illustrates the difference of pro- and mesotarsus between *Graphoderus perplexus* and *Graphoderus elatus*.

**Table 1.** Results of independent 2-group Mann-Whitney tests. P-values for ratios and the average number of adhesive discs on pro- and mesotarsus between *Graphoderus perplexus* and *Graphoderus elatus*, representing the number of specimens in each test, and representing the outcome value from each test (**W**).

Ratio or tarsus	W	*Graphoderus perplexus*	*Graphoderus elatus*	P-value
PL/TL	901	57	16	< 0.001
PW/PL	55	57	16	< 0.001
PLLL/PCLL	475	57	16	0.8051
PCLWt/PCLWb	500	56	15	0.2628
Average protarsus	210	17	14	< 0.001
Average mesotarsus	195	16	13	< 0.001

No statistical significance were found in the ratios PLLL/PCLL (relative extension of central penis lobe to lateral lobes, Mann-Whitney test, P = 0.8051) or in PCLWt/PCLWb (anterior narrowing of central penis lobe, Mann-Whitney test, P = 0.2628) (Table 1). The four genitalia ratio tests were conducted using Bonferroni correction to adjust alpha to 0.0125 (0.05/4). The results clearly reject the hypothesis that *Graphoderus perplexus* and *Graphoderus elatus* are the same species.

## Taxonomy

### Graphoderus elatus

Taxon classificationAnimaliaColeopteraDytiscidae

Sharp, 1882 new status

Graphoderus elatus Sharp, 1882: 695 (original description);
Graphoderus cinereus sensu Horn (1883) (in part);
Graphoderus zonatus sensu Zimmermann (1920) (in part), Gschwendtner (1937) (in part), Kamiya (1940), Balfour-Browne (1946), Zaitsev (1972), Lafer (1989), Zeng (1989), Mori and Kitayama (1993), Nilsson (1995), Nilsson et al. (1997);
Graphoderus zonatus zonatus sensu Nilsson (2003a) (in part), Nilsson and Hájek (2015) (in part);
Graphoderus zonatus verrucifer sensu Nilsson et al. (1997), Nilsson (2003a) (in part), Nilsson and Hájek (2015) (in part);
Graphoderus perplexus sensu Wallis (1939) (in part), Larson (1975) (in part), Nilsson et al. (1999), Nilsson (2001) (in part), Nilsson (2003a) (in part), Nilsson (2015) (in part), Nilsson and Hájek (2015) (in part).

#### Type locality.

Russia > East Siberia > Amurland.

#### Type material.

Lectotype ♂ (BMNH), by present designation. Labeled: “Eastern Siberia 995 elatus. Sharp Coll. 1905-313. Data in NHRS JLKB 000023379. Lectotype *Graphoderus elatus* Sharp, 1882 Des. S. Holmgren et al., 2015”. Paralectotype ♂ (BMNH). Labeled: “Red River. Am. Bor. 995 var. Paralectotype. Sharp Coll. 1905-313. Data in NHRS JLKB 000023380. *Graphoderus perplexus* Sharp 1882 Det. J. Bergsten, 2015”.

#### Lectotype justification.

**Figure 7.**
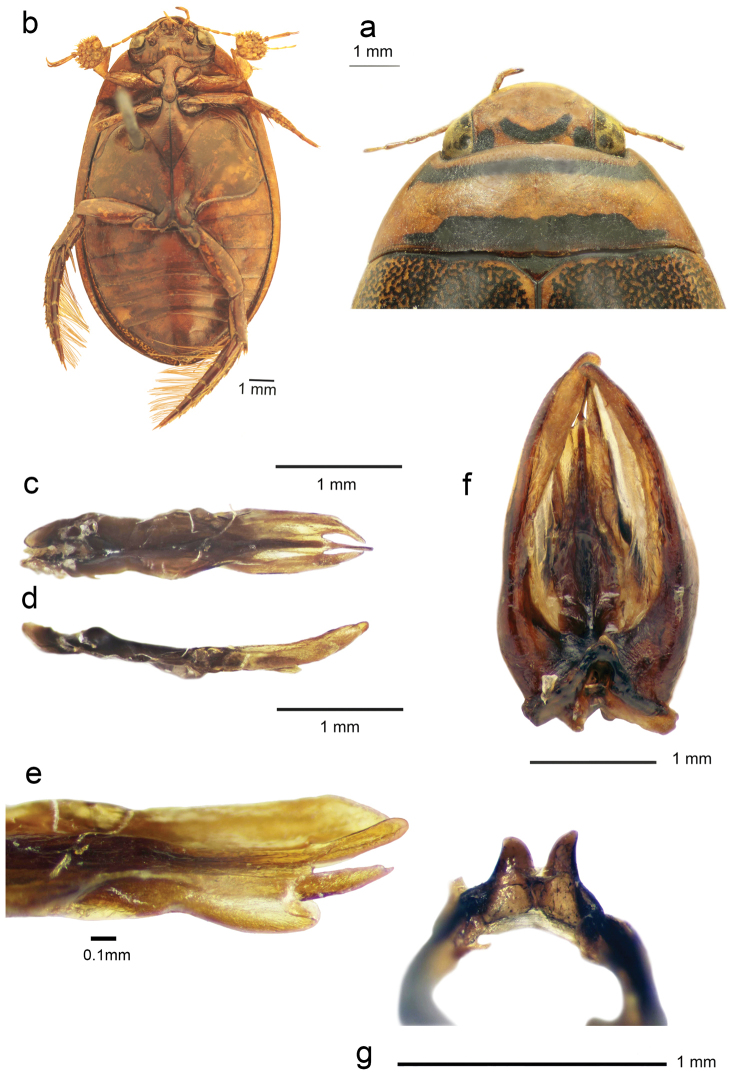
The designated lectotype for *Graphoderus elatus* Sharp, 1882 (BMNH). **a** head and pronotum (2.1 mm long, maximum width 6.5 mm) in dorsal view **b** ventral view (body length 14.0 mm) **c** penis in dorsal view (2.8 mm, long 0.55 mm width) **d** penis in lateral view **e** central penis lobe with concave dorsal margin **f** entire genitalia with parameres surrounding the penis **g** lappets of aedeagal ring.

Sharp (1882) based his description of *Graphoderus elatus* on two male specimens, one from “Eastern Siberia (Amurland)” and the other from “North America (Red River)”. He gave *Graphoderus elatus* the number 995. These two syntype specimens, present in Sharp’s collection (BMNH), were studied and genitalia extracted. Both are pinned through cards, these cards being mounted on longer pins. The Siberian specimen is labeled on the face of the card, in Sharp’s handwriting “Eastern Siberia 995 elatus”, while the North American specimen is labeled “Red River. Am. Bor. 995 var”. The syntype specimen from Red River Am. Bor. we consider conspecific with *Graphoderus perplexus*. The Lectotype of *Graphoderus perplexus* (designated by Larson 1975) was studied at BMNH but is a female so the shape of male genitalia could not be compared. The characters Sharp (1882) used to distinguish *Graphoderus elatus* from *Graphoderus perplexus* are unconvincing as already argued by Wallis (1939). The syntype specimen from Siberia has the concave outline of the penis’ apex central lobe in lateral view. The penis is also of the longer type (Fig. 2k) and belongs to what we initially called *Graphoderus perplexus*-like specimens from the east Palearctic. We therefore designate the Siberian specimen as lectotype of *Graphoderus elatus* Sharp, 1882, and have so labeled it. What we initially called *Graphoderus perplexus*-like specimens from the east Palearctic belong to *Graphoderus elatus* which is here reinstated as a valid species (stat. n.). The American specimen although belonging to *Graphoderus perplexus*, is a paralectotype of *Graphoderus elatus*. As Sharp’s description of *Graphoderus elatus* is very short and was based on a mix of two species we provide a redescription and documentation (Fig. 7) based on the designated lectotype followed by a discussion of the intraspecific variations as here interpreted from all examined material (Table 2).

**Table 2.** Studied material of *Graphoderus elatus*. Sex, catalog number (ID), deposition, locality information, latitude (Lat.), longitude (Lon.), date collected and collector of the studied *Graphoderus elatus* specimens. ♂=male, ♀=female.

Sex	Catalog ID	Museum	Locality	Lat.	Lon.	Date	Collectors
♂†	NHRS-JLKB000023379	BMNH	Russia, Amurland, Siberia				
♂	NHRS-JLKB000040578	NHRS	Russia, Kamchatka, Ponds inland from the bay between Cape Zheltyi (south) and Cape Ilya (north)	51.5583°N	157.709°E	1999-07-27	Minakawa & Kurowski
♂	NHRS-JLKB000040579	NHRS	Russia, Kamchatka, Ponds inland from the bay between Cape Zheltyi (south) and Cape Ilya (north)	51.5583°N	157.709°E	1999-07-27	Minakawa & Kurowski
♀	NHRS-JLKB000040580	NHRS	Russia, Kamchatka, Ponds inland from the bay between Cape Zheltyi (south) and Cape Ilya (north)	51.5583°N	157.709°E	1999-07-27	Minakawa & Kurowski
♀	NHRS-JLKB000040581	NHRS	Russia, Kamchatka, Ponds inland from the bay between Cape Zheltyi (south) and Cape Ilya (north)	51.5583°N	157.709°E	1999-07-27	Minakawa & Kurowski
♀	NHRS-JLKB000040582	NHRS	Russia, Kamchatka, Ponds inland from the bay between Cape Zheltyi (south) and Cape Ilya (north)	51.5583°N	157.709°E	1999-07-27	Minakawa & Kurowski
♂	NHRS-JLKB000000954	NHRS	Russia, Kamchatka, Ponds inland from the bay between Cape Zheltyi (south) and Cape Ilya (north)	51.5583°N	157.709°E	1999-07-27	Minakawa & Kurowski
♂	NHRS-JLKB000040583	NHRS	Russia, Kamchatka, Elizovo, 12km S	53.0283°N	158.6454°E	1997-07-09	Kholin
♂	NHRS-JLKB000040584	NHRS	Russia, Kamchatka, Elizovo, 12km S	53.0283°N	158.6454°E	1997-07-09	Kholin
♂	NHRS-JLKB000040591	NHRS	Russia, Kamchatka, Elizovo, 12km S	53.0283°N	158.6454°E	1997-07-09	Kholin
♀	NHRS-JLKB000040585	NHRS	Russia, North Sakhalin, Val river env.	52.493°N	142.683°E	2002-07-29	Minakawa
♂	NHRS-JLKB000040586	NHRS	Russia, North Sakhalin, Val river env.	52.493°N	142.683°E	2002-07-29	Minakawa
♀	NHRS-JLKB000040587	NHRS	Russia, Lopukhovaya, Urup, Kuril islands	45.7965°N	149.9002°E	1995-08-29	Oberg
♂	NHRS-JLKB000040588	NHRS	Russia, Lopukhovaya, Urup, Kuril islands	45.7965°N	149.9002°E	1995-08-29	Oberg
♂	NHRS-JLKB000040589	NHRS	Russia, Lopukhovaya, Urup, Kuril islands	45.7965°N	149.9002°E	1995-08-28	Oberg
♂	NHRS-JLKB000040590	NHRS	Russia, Lopukhovaya, Urup, Kuril islands	45.7965°N	149.9002°E	1995-08-28	Oberg
♀	NHRS-JLKB000023362	NHRS	Japan, Horonobe-chô, Teshio gun, Hokkaido	45.0172°N	141.8491°E	1999-10-30	Kamite
♂	NHRS-JLKB000023363	NHRS	Japan, Horonobe-chô, Teshio gun, Hokkaido	45.0172°N	141.8491°E	1999-10-30	Kamite
♀	NHRS-JLKB000000961	NHRS	Japan, Horonobe-chô, Teshio gun, Hokkaido	45.0172°N	141.8491°E	2009-09-13	Nakajima
♂	NHRS-JLKB000023364	NHRS	Russia, Shimanovsk, Amur region	52.0011°N	127.6842°E	1975-06-20 - 29	Zolotukhin
♂	NHRS-JLKB000000951	ZMUM	Russia, Lake Kenon, Chita region	52.0402°N	113.3856°E	1973-08-06	Berlov
♂	NHRS-JLKB000000952	ZMUM	Russia, Lake Kenon, Chita region	52.0402°N	113.3856°E	1971-08-06	Berlov
♂	NHRS-JLKB000000953	ZMUM	Russia, Lake Kenon, Chita region	52.0402°N	113.3856°E	1973-08-06	Berlov
♀	NHRS-JLKB000023365	OMNH	Japan, Wakasakanai, Toyotomi	45.1059°N	141.6328°E	1987-08-01	Mori
♂	NHRS-JLKB000023369	OMNH	Japan, Wakasakanai, Toyotomi	45.1059°N	141.6328°E	1987-07-31	Mori
♀	NHRS-JLKB000023370	OMNH	Japan, Wakasakanai, Toyotomi	45.1059°N	141.6328°E	1987-07-31	Mori
♂	NHRS-JLKB000023366	OMNH	Japan, Wakasakanai, Toyotomi	45.1059°N	141.6328°E	1993-07-25	Hayashi
♂	NHRS-JLKB000023367	OMNH	Japan, Wakasakanai, Toyotomi	45.1059°N	141.6328°E	1993-07-25	Hayashi
♀	NHRS-JLKB000023368	OMNH	Japan, Wakasakanai, Toyotomi	45.1059°N	141.6328°E	1993-07-25	Hayashi
♂	NHRS-JLKB000023371	OMNH	Japan, Bakkaimura, Yuukuru	45.3103°N	141.6207°E	1992-08-22	Kitayama
♂	NHRS-JLKB000023372	OMNH	Japan, Sarobetsu, Wakasakanai	45.0853°N	141.8197°E	1992-08-21	Kitayama
♀	NHRS-JLKB000023373	OMNH	Japan, Sarobetsu, Wakasakanai	45.0853°N	141.8197°E	1992-08-21	Kitayama
♀	NHRS-JLKB000023374	OMNH	Japan, Sarobetsu, Wakasakanai	45.0853°N	141.8197°E	1992-08-21	Kitayama
♀	NHRS-JLKB000023375	BMNH	Russia, Yakutsk, 18 km E of river Lena, Siberia	61.4372°N	131.0155°E	1970-07-21	Angus
♂	NHRS-JLKB000023376	BMNH	Russia, Yakutsk, 18 km E of river Lena, Siberia	61.4372°N	131.0155°E	1970-07-21	Angus
♂	NHRS-JLKB000023377	BMNH	China, Gangca, Qinghai Hu, Qinghai N	37.2952°N	100.1797°E	2013-06-05	Angus, Jia & Zhang
♂	NHRS-JLKB000023378	BMNH	China, Gangca, Qinghai Hu, Qinghai N	37.2952°N	100.1797°E	2013-06-05	Angus, Jia & Zhang
♂		BMNH	China, Gangca, Qinghai Hu, Qinghai N	37.2952°N	100.1797°E	2013-06-05	Angus, Jia & Zhang
♂		BMNH	China, Gangca, Qinghai Hu, Qinghai N	37.2952°N	100.1797°E	2013-06-05	Angus, Jia & Zhang
♂		SYSU	China, Gangca, Qinghai Hu, Qinghai N	37.2952°N	100.1797°E	2013-06-05	Angus, Jia & Zhang
♂		SYSU	China, Gangca, Qinghai Hu, Qinghai N	37.2952°N	100.1797°E	2013-06-05	Angus, Jia & Zhang
♀		BMNH	China, Gangca, Qinghai Hu, Qinghai N	37.2952°N	100.1797°E	2013-06-05	Angus, Jia & Zhang
♀		SYSU	China, Gangca, Qinghai Hu, Qinghai N	37.2952°N	100.1797°E	2013-06-05	Angus, Jia & Zhang
♂		SYSU	China, Gangca, Qinghai Hu, Qinghai N	37.2952° N	100.1797° E	2013-06-05	Angus, Jia & Zhang
♂	NHRS-JLKB000023381	ZIN	Mongolia, Onon river	48.5941°N	110.8558°E	1987-08-29	Dulma
♂	NHRS-JLKB000023382	ZIN	Mongolia, Onon river	48.5941°N	110.8558°E	1987-08-29	Dulma
♂	NHRS-JLKB000023383	ZIN	Russia, Indigirka river	69.5267°N	146.6575°E	1891-07-16	Cherskiy
♂		ZIN	Russia, Indigirka river	69.5267°N	146.6575°E	1891-07-16	Cherskiy
♂		ZIN	Russia, Indigirka river	69.5267°N	146.6575°E	1891-07-16	Cherskiy
♂	NHRS-JLKB000023384	ZIN	Russia, Verkhoyansk	67.8181°N	134.0181°E	1885-05 & 07	Bung & Tol.
♂		BMNH	China, Lesser Kingan, Mts China	49.0892°N	127.5374°E		Weymarn
♂		BMNH	China, Lesser Kingan, Mts China	49.0892°N	127.5374°E		Weymarn
♀		SYSU	China, Nei Mongol, Hulunber, Huihe			2013-07-22	Li, Chunyuan & Chaoqun
♂		SYSU	China, Inner Mongolia (Nei Mongol), Xing’an near entry-exit inspection of border between China and The Republic of Mongolia			2014-07-24	Jia
♀		SYSU	China, Inner Mongolia (Nei Mongol), Xing’an near entry-exit inspection of border between China and The Republic of Mongolia			2014-07-24	Jia

† Lectotype.

#### Description of the Lectotype ♂.

Body length 14.0 mm; maximum elytral width 8.3 mm.

Head (Fig. 7a) dorsally testaceous; basal black band extending between eyes, retracted under anterior of pronotum, extending apically to eyes with no apparent separation from eyes; V-shaped black marking anteriorly. Head ventrally testaceous; clypeus testaceous; maxillary palpi yellow with apical palpomere piceous distally. Labial palpi yellow with apical palpomere darkened along its inner margin. Antenna testaceous with antennomeres more or less piceous in distal half.

Pronotum (Fig. 7a) 2.1 mm long; maximum width 6.5 mm; testaceous; transverse black bands separated from anterior margin by testaceous band and from posterior margin by narrow testaceous, more piceous band; black bands do not reach sides of pronotum; anterior black band at edges with narrow posteriorly directed projections; posterior black band with narrow lateral portions projecting from basal margin of band. Elytron 10.5 mm long; smooth, yellow with black irrorations; irrorations reduced along margins of elytra; sutural midline black; scutellum piceous.

Ventral side (Fig. 7b) testaceous-rufous, darkened due to age so that minor variations in its color not apparent. Forelegs testaceous; midlegs partly testaceous, mesotibia and mesotarsus rufous-testaceous with golden setae along edge; metatrochanter and metafemur testaceous; metatibia rufous with golden setae along edge; metatarsus rufous-piceous with long golden setae along edge. Elytral epipleuron testaceous; broad anteriorly, gradually tapering along edges of abdomen but relatively broad also in first part of posterior half.

Protarsal claws similar in size and shape, shorter than protarsomere V; mesotarsal claws similar in size and shape. Posterior metatarsal claws almost three times as long as anterior metatarsal claws. Protarsomeres I-III enlarged with three larger adhesive discs basally and about 32 smaller discs distally. Mesotarsomeres with two more or less regular rows of seven discs, left mesotarsus with one additional smaller disc on mesotarsomere I and a second on mesotarsomere II and right one with only one extra disc, on mesotarsomere II.

Penis in dorsal view (Fig. 7c) about 2.8 mm long; width 0.55 mm; apex trifid with three distinct lobes. Penis in lateral view (Fig. 7d) with lateral apical lobes fairly slender; central penis lobe sclerotized along edge, longer than side lobes, its dorsal margin concave (Fig. 7e). Parameres with external margins straighter medially, their apices convergent (Fig. 7f). Lappets of aedeagal ring sclerite short and wide, their outer apical margins rounded (Fig. 7g).

#### Intraspecific variation.

Body length between 13.9 and 16.3 mm; maximum elytral width between 8.0 and 9.6 mm. Pronotum length 1.8 to 2.5 mm long; width 5.3 to 7.2 mm; smooth in males; either deeply wrinkled (when also elytra granulated) or smooth in females; in smooth specimens either shining with anterior row of impressed punctures very distinct, or matt with puncture-row less distinct; anterior black band of pronotum mostly continuous, sometimes thin and weak or non-continuous; shape of posterior black band of pronotum varies, separated from posterior margin by testaceous band which sometimes is partly piceous. Elytron between 10.4 and 12.7 mm long; smooth in males; smooth or granulated in females.

Male posterior metatarsal claws almost three times as long as anterior metatarsal claws; female posterior metatarsal claws less than twice as long as anterior metatarsal claws which are slightly curved apically. Protarsomeres I-III enlarged in males with three larger adhesive discs basally and 28-66 smaller discs distally; mesotarsomeres in males with irregular rows of 14-31 adhesive discs; in populations with granulated females, number of adhesive discs in males are in upper range. Penis in dorsal view between 2.4 and 2.8 mm long; width between 0.6 and 0.7 mm. Shape of lappets in aedeagal ring sclerite variable which also applies to outer apical margin.

#### Distribution.

**Figure 8.**
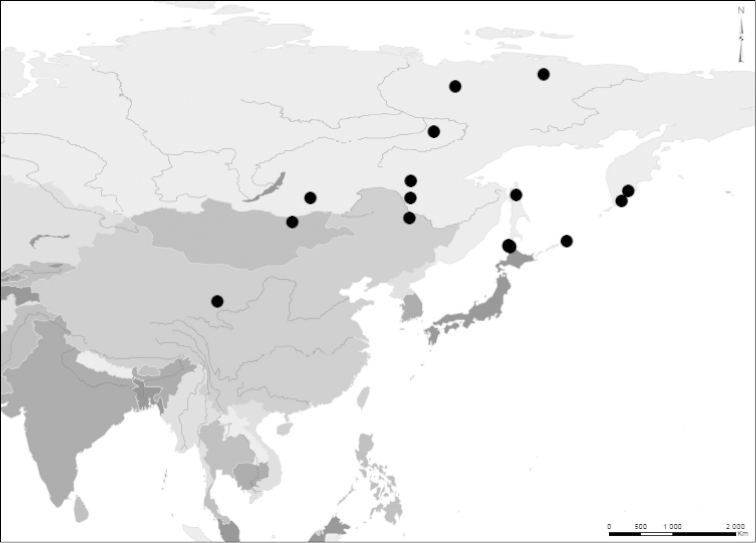
Distribution map of *Graphoderus elatus* based on examined specimens. The lectotype is imprecisely marked in former Amur Region.

(Fig. 8) The distribution covers Russia, east of the Yenisei-Angara river to the pacific coast, north to the East Siberian Sea and south to Qinghai in China and Hokkaido in Japan. Specimens from the following regions in east Palearctic were examined: Russia: Yakutia, Kamchatka, Chita Region, Amur Region, Kuril Islands, North Sakhalin, Verkhoyansk and Indigirka. Japan: Hokkaido. Mongolia: Onon river. China: Heilongjiang, Inner Mongolia and Qinghai.

### Key to *Graphoderus* species

As the resolved situation in the east Palearctic means that there are no species in common between Nearctic and Palearctic the key is constructed with a first dichotomy between the continents for ease of use. In order for both males and females to be identifiable, each key step has multiple characters and characters of the pronotal black bands are included as they are often very useful albeit not always absolutely trustworthy. Mesotarsal formula, e.g. 6-4-4, refers to six adhesive discs on mesotarsomere I, four on mesotarsomere II and four on mesotarsomere III. Note that there are errors in the *Graphoderus* genitalia figured in Larson et al. (2000); 190c labeled as *Graphoderus perplexus* is more likely *Graphoderus fascicollis* and 190b labeled as *Graphoderus fascicollis* is possibly *Graphoderus manitobensis*, and true *Graphoderus perplexus* genitalia does not seem to be included in the figure.

**Table:** 

1	Nearctic species	**2**
–	Palearctic species	**6**
2	Head and pronotum yellow to reddish brown with no defined black markings (Fig. 9h); body length 10.4 to 12.4 mm; male genitalia simple with barely trifid apex (Fig. 2o–p)	***Graphoderus liberus***
–	Head with black V-shaped markings and pronotum with two well-defined black bands (Fig. 9g, i–k); body length larger, up to 15.7 mm; penis with distinct trifid apex (Fig. 2m–n, q–v)	**3**
3	Posterior black band of pronotum not reaching posterior margin, or sometimes separated from margin by a piceous-reddish area, anterior black band separated from anterior margin (Fig. 9k); male protarsus with 25–35 adhesive discs, male mesotarsus with 13–20 discs; male penis deeply trifid, invaginations separating lateral lobes from central lobe distinctly deeper than width of lateral lobes of penis apex, as in Figure 2u–v	***Graphoderus perplexus***
–	Posterior black band of pronotum contiguous with posterior margin, anterior black band of pronotum contiguous or not with anterior margin; male tarsal discs various, mesotarsus with 0, 12 or 25–30 discs; trifid apex of male penis shallower, invaginations not deeper than width of lateral lobes of penis apex (Fig. 2m–n, q–t)	**4**
4	Anterior black band of pronotum contiguous with anterior margin (Fig. 9j); female pronotum with weak corrugated sculpture; male mesotarsus not dilated and lacking adhesive discs; male protarsal claws different in shape and size, posterior claw with sinuate ventral margin and about 2/3 length of anterior claw; male parameres very long, at least 1/4th longer than penis (Fig. 2s)	***Graphoderus occidentalis***
–	Anterior black band of pronotum mostly separated from anterior margin by a more or less evident reddish area; female pronotum with conspicuous corrugated sculpture; male mesotarsus dilated with adhesive discs on ventral surface; male protarsal claws equal or anterior claw only slightly longer than posterior which does not have a sinuate ventral margin; male parameres shorter, not more than 1/5th longer than penis (Fig. 2m, q)	**5**
5	Metanepisterna (“metasternal wing”) broad, width between 0.48 and 0.60 mm; female elytron at shoulder with less pronounced striolate punctures; male mesotarsus with 12 discs in two rows; central penis lobe of trifid apex much shorter than lateral lobes (Fig. 2m–n)	***Graphoderus fascicollis***
–	Metanepisterna (“metasternal wing”) narrower, width between 0.30 and 0.41 mm; female elytron at shoulder with pronounced strioles; male mesotarsus with 25–30 discs in four rows; central penis lobe of trifid apex about as long as lateral lobes (Fig. 2q–r)	***Graphoderus manitobensis***
6	Posterior black band of pronotum narrow, equal to only 1/3 to 1/2 of medial yellow area, contiguous with posterior margin (Fig. 9d); epipleuron broader at level of abdominal ventrite II than at level of ventrite I, body “pear-shaped” due to posteriorly widened epipleura; male genitalia as in Figure 2g–h	***Graphoderus bilineatus***
–	Posterior black band of pronotum broad, equal to at least 1/2 of medial yellow area, or if narrower then not contiguous with posterior margin; epipleuron evenly tapering from base to apex, body not overly “pear-shaped”	**7**
7	Ventral side of body mostly piceous; metatibia and metatarsus dark brown to black; female pronotum with conspicuous corrugated sculpture; anterior black band of pronotum continuous with anterior margin, in males this band is narrow and equal to about 1/3 of medial yellow band (Fig. 9a); male genitalia as in Figure 2a–b. East Palearctic	***Graphoderus adamsii***
–	Ventral side of body testaceous-rufous, sometimes piceous but then entire habitus darker; female pronotum with or without conspicuous corrugated sculpture; anterior black band of pronotum continuous or not with anterior margin, if continuous in males broader then 1/3 of medial yellow band. East or west Palearctic	**8**
8	Transverse black bands of pronotum contiguous with anterior and posterior margin, respectively (Fig. 9b); anterior mesotarsal claw longer than posterior claw, strongly in males weakly in females; female pronotum with weak corrugated sculpture; male mesotarsus not dilated and without discs; trifid apex of male penis very shallow, parameres very long, at least 1/4th longer than penis (Fig. 2c–d)	***Graphoderus austriacus***
–	Anterior and posterior black bands of pronotum contiguous or not with margins; mesotarsal claws of same length in both sexes; female pronotum corrugated or not; male mesotarsus dilated and with adhesive discs on ventral surface; male penis apex moderate to deeply trifid and parameres shorter, maximum 1/5th longer than penis (Fig. 2e–f, i–l, w–x)	**9**
9	Transverse black bands of pronotum not contiguous with anterior and posterior margin, separated by narrow bands or rarely almost contiguous; female elytra granulated or not; male mesotarsus with 14–60 adhesive discs that are small and usually in irregular rows	**10**
–	Posterior black band of pronotum contiguous with posterior margin, anterior transverse band contiguous with anterior margin or narrowly separated by rufous area (Fig. 9e); female elytra never granulated; male mesotarsus with 12–14 discs that are larger and in two regular rows	**11**
10	Epipleura rather wide at level of abdominal ventrites I-III (Fig. 10f–h); central lobe of male trifid apex in lateral view concave (Fig. 2l). East Palearctic, east of Yenisei-Angara river	***Graphoderus elatus***
–	Epipleura narrower at level of abdominal ventrites I-III (Fig. 10a–b); central lobe of male trifid apex in lateral view convex (Fig. 2x). Palearctic, west of Yenisei-Angara river	***Graphoderus zonatus***
11	Minimum distance between meso- and metacoxae almost same as width of metaventral process between mesocoxae (Fig. 11a); female posterior metatarsal claw about 1.8 the length of anterior claw, which is not strongly curved apically; male mesotarsus with 12 discs in two rows ventrally, formula 4-4-4; male penis apex less deeply trifid and central lobe in lateral view more abruptly raised (Fig. 2e–f). East Palearctic	***Graphoderus bieneri***
–	Minimum distance between meso- and metacoxae clearly less than width of metaventral process between mesocoxae (Fig. 11b); female posterior metatarsal claw about 1.5 the length of anterior claw which is strongly curved apically; male mesotarsus with 14 discs in two rows, formula 6-4-4; male penis deeply trifid and central lobe in lateral view describing a long evenly convex curve (Fig. 2i–j). Palearctic	***Graphoderus cinereus***

**Figure 9.**
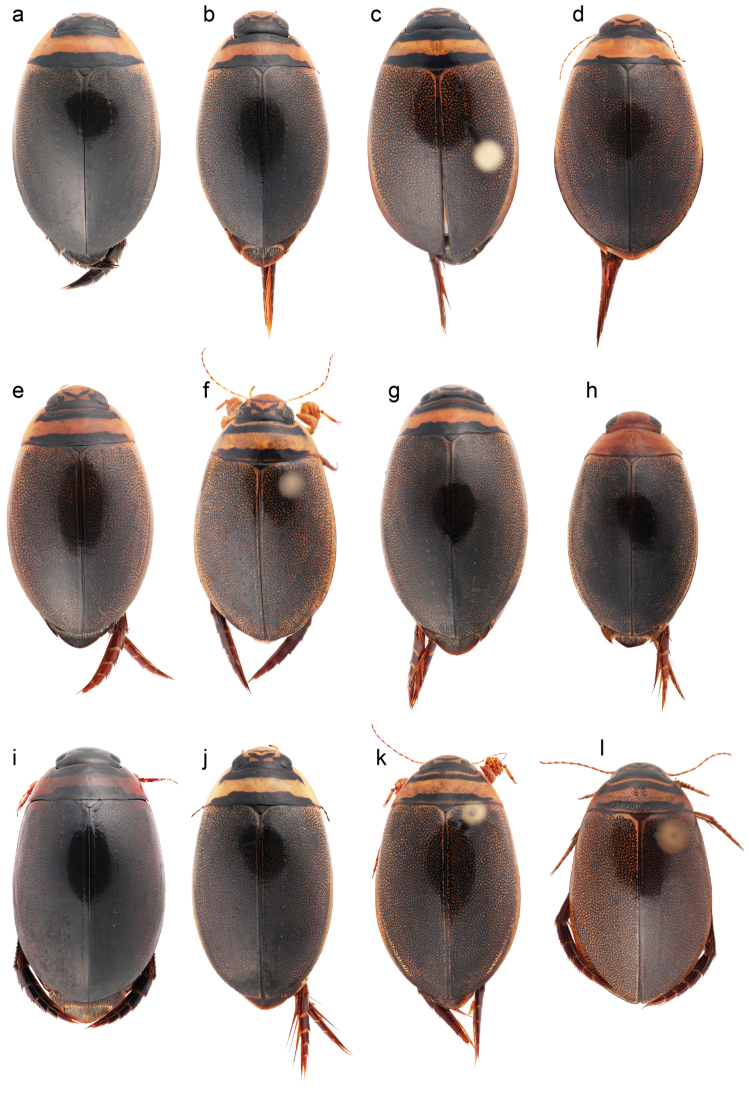
Habitus photographs of all *Graphoderus* species in dorsal view. **a** *Graphoderus adamsii* **b** *Graphoderus austriacus* **c** *Graphoderus bieneri* **d** *Graphoderus bilineatus* **e** *Graphoderus cinereus* **f** *Graphoderus elatus* **g** *Graphoderus fascicollis* **h** *Graphoderus liberus* **i** *Graphoderus manitobensis* **j** *Graphoderus occidentalis* **k** *Graphoderus perplexus* **l** *Graphoderus zonatus*.

**Figure 10.**
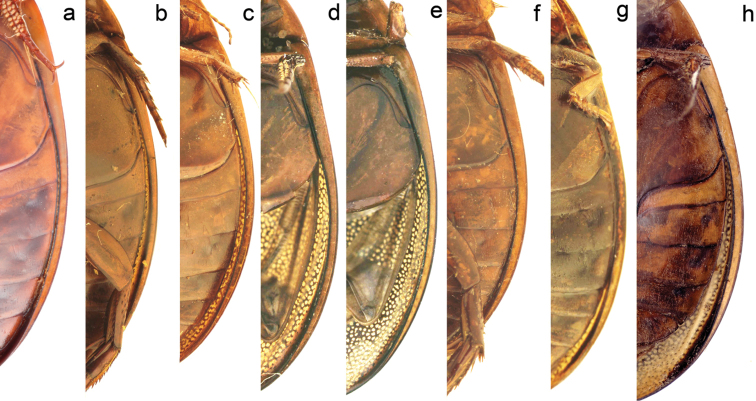
Ventral view showing the epipleural width. **a–b** *Graphoderus zonatus* **c–e** *Graphoderus perplexus* and **f–h** *Graphoderus elatus*. Specimens from Sweden (**a**), France (**b**), USA (**c** Lectotype of *Graphoderus perplexus*), Canada, Quebec (**d**), Red River Am. Bor. (**e** paralectotype of *Graphoderus elatus*), Amurland Russia (**f** Lectotype *Graphoderus elatus*), Gangca China (**g**), “Manchuria” Weymarn coll. (**h**). The species differ in the epipleural width especially at level of abdominal ventrites I-III.

**Figure 11.**
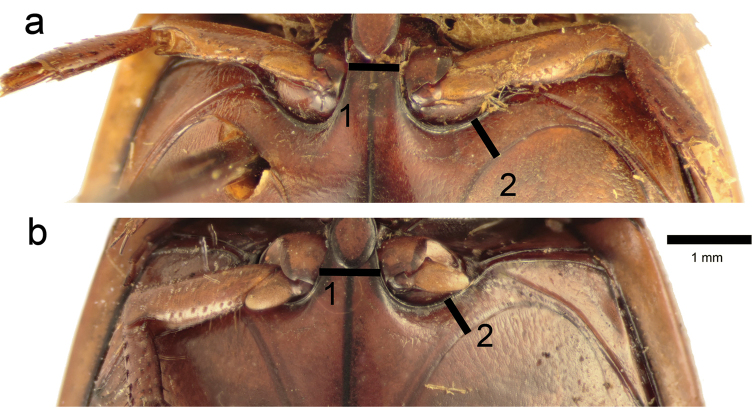
Ventral view showing meso- and metathorax of *Graphoderus bieneri* (**a**) and *Graphoderus cinereus* (**b**). To indicate the relative distances between the mesocoxae (1) and the meso- and metacoxae (2).

## Discussion

The within species variation in the shape and extension of the transverse black bands on the pronotum in *Graphoderus zonatus* (Nilsson 1986) was observed to exist in *Graphoderus perplexus* and *Graphoderus elatus* as well. Rarely, *Graphoderus zonatus* specimens are found where the basal black band reaches all the way to the posterior margin. Initially, we were struck by the very thin black bands in several specimens of *Graphoderus elatus*, and while this color pattern seems more common in *Graphoderus elatus* than in either *Graphoderus zonatus* or *Graphoderus perplexus* we have in some specimens found the same coloration in the latter species too. We therefore consider this character as less reliable for species diagnosis within the *zonatus*-species complex. We also examined the shape of the bifurcation in the chitinous ring around the male genitalia, which Wallis (1939) indicated as diagnostic. We found it to be informative, probably significantly so if the shape had been quantified and tested statistically but we observed within species variation in all three species. It has also been seen in *Graphoderus zonatus* that the coloration of elytra can probably be connected to the habitat e.g. darker specimens have more often been found in dystrophic water (Nilsson 1986) and this might indicate that specific coloration may also be an inferior character to separate species. Instead the great interspecific variation in the male genitalia, especially at the top of the penis, is commonly used to separate species within Dytiscidae (Nilsson and Holmen 1995, Larson et al. 2000, Miller 2001, Bergsten and Miller 2006), and we found this as the most informative character (Fig. 2). The shape in lateral view of the central lobe at the penis’ trifid apex was a qualitative non-overlapping character we found the most reliable to separate *Graphoderus zonatus* from *Graphoderus perplexus*/*Graphoderus elatus*. The male penis (and parameres) were significantly longer relative to body length and also had a more elongate shape as measured by a length to width ratio in *Graphoderus elatus* compared to *Graphoderus perplexus*. Though significant, variation was overlapping for these characters (Fig. 4). Although not quantified, the epipleura are also in general wider in *Graphoderus elatus* not only compared to *Graphoderus zonatus*, but also compared to *Graphoderus perplexus* (Fig. 10).

Since *Graphoderus elatus* was described by Sharp (1882) it has mostly been treated as a synonym of different species (Horn 1883, Zimmermann 1917, Wallis 1939) rather than a valid species. Wallis (1939) was the one who concluded that its diagnostic character was not enough to retain both *Graphoderus perplexus* and *Graphoderus elatus*, but neither the size nor the shape of the male genitalia had been considered (Sharp 1882, Wallis 1939). However, when Nilsson et al. (1999) later identified five adult specimens from Kamchatka as *Graphoderus perplexus*, they somewhat misleadingly stated this to be the first Palearctic record of the species despite the fact that in synonymizing *Graphoderus elatus* with *Graphoderus perplexus* Wallis (1939) defined *Graphoderus perplexus* as a species occurring in both Nearctic and east Siberia of Russia. It was not until the compilation of the world catalogue two years later that Nilsson (2001) brought back the somewhat forgotten name *Graphoderus elatus*. As Nilsson’s collection now is housed at the NHRS, we have re-examined all *Graphoderus* material from Kamchatka (Nilsson et al. 1999) and Urup, Kuril Islands (Nilsson et al. 1997) as well as material from North Sakhalin collected by Minakawa in 2002. All the males proved to have the concave shape of the central penis lobe that separates *Graphoderus elatus* and *Graphoderus perplexus* from *Graphoderus zonatus* (Fig. 2) and the genitalia also proved to be larger than *Graphoderus perplexus*, fitting into the diagnosis of *Graphoderus elatus*.

From our results we here propose a strict allopatric distribution of the three species in the *zonatus* complex. We propose that 1) *Graphoderus perplexus* only occurs in the Nearctic region, 2) *Graphoderus elatus* only occurs in the east Palearctic region, east of the Yenisei-Angara river and 3) *Graphoderus zonatus* occurs in the Palearctic region from central and north Europe through Turkey, Caucasus and eastwards up to the Yenisei-Angara river. It is likely that there is a contact zone where *Graphoderus zonatus* and *Graphoderus elatus* meet, but whether any hybridization occurs is unknown. All *Graphoderus zonatus* records from Amur, Chita, Verkhoyansk, Indigirka, Kamchatka, Sakhalin and Kuril Islands are *Graphoderus elatus*. Also material from Magadan, Khabarovsk and Primorsky Kray reported by Lafer (1989) is most likely *Graphoderus elatus* (not examined). Material we have examined from Yakutia has also been *Graphoderus elatus* and records referred to East Siberia are probably mostly *Graphoderus elatus* but could refer to *Graphoderus zonatus*. The material we studied from northeast Mongolia was *Graphoderus elatus* and the reported *Graphoderus zonatus* from the north-central and western part of the country (Shaverdo et al. 2008) might be misidentified *Graphoderus elatus*. Likewise, the newly collected material from Gangca in China was *Graphoderus elatus* as well as material from Heilongjiang and Inner Mongolia, but *Graphoderus zonatus* may occur in the more western parts of China. What was thought to be *Graphoderus zonatus* in Japan was first discovered on Hokkaido by Matsumoto in 1986 (Mori and Kitayama 1993). We here conclude that all material from Japan referred to *Graphoderus zonatus* is instead *Graphoderus elatus*.

Our easternmost record of true *Graphoderus zonatus* was collected outside Irkutsk, just west of the Yenisei-Angara river. Strikingly this is also the easternmost record of several other west Palearctic aquatic beetles like *Helophorus granularis* (Linnaeus, 1761), *Helophorus strigifrons* Thomson, 1868 and *Helophorus pumilio* Erichson, 1837 (Angus 2011). The sister species pair in the *Agabus lineatus* Gebler, 1848 species group is also separated east and west of Yenisei-Angara river (Nilsson 2003b). As well, the Palearctic *Colymbetes paykulli* Erichson, 1837 and the Holarctic *Colymbetes dahuricus* Aubé, 1837 are separated in this area (Drotz et al. 2015). All other examined “*Graphoderus zonatus*” from the Palearctic, east of the Yenisei turned out to be *Graphoderus elatus* but it is important to note that the number of adhesive discs on male pro- and mesotarsus are not reliable characters to separate *Graphoderus zonatus* from *Graphoderus elatus*. In the populations of *Graphoderus elatus* from Urup, Inner Mongolia (leg. Li, Chunyuan and Chaoqun) and Yakutsk (18 km E. of river Lena) the females had granulated elytra and the males from Urup had a significant larger number of adhesive discs. In the material examined from Kamchatka, Hokkaido, Inner Mongolia (leg. Jia), Gangca and Heilongjiang females had smooth elytra and males a lower number of adhesive discs. Yet our examined material from North Sakhalin indicates that the two morphs can exist within the same population. However, the question arises whether these dorsal female forms could have arisen twice, completely independent of each other or if they are sister-species and inherited the polymorphism from a common ancestor. Based on the male genitalia it seems more likely that *Graphoderus elatus* and *Graphoderus perplexus* are sister-species, which would require either a loss of the polymorphism in *Graphoderus perplexus* or independent gains in *Graphoderus zonatus* and *Graphoderus elatus*. We are inclined to believe that a loss of polymorphism is more likely than independent gains, but phylogenetic studies (in progress) are first required to establish sister-species relationship in *Graphoderus*.

## Conclusions

From qualitative and quantitative characters of the male genitalia the earlier synonymized name *Graphoderus elatus* Sharp, 1882 is reinstated as a valid species and a lectotype has been designated. We propose an allopatric distribution of species in the *zonatus*-species complex where *Graphoderus perplexus* is Nearctic, *Graphoderus elatus* only occurs in east Palearctic, east of the Yenisei-Angara river and *Graphoderus zonatus* occurs only west of the same river. All previous *Graphoderus zonatus* records from east of this river are misidentified *Graphoderus elatus*. Surprisingly, there are now two *Graphoderus* species with distinctly dimorphic females, *Graphoderus zonatus* and *Graphoderus elatus*. Finally, by providing male genitalia illustrations together with a new identification key to all *Graphoderus* species we hope to aid future identification work and taxonomic endeavors in the group.

## Supplementary Material

XML Treatment for Graphoderus elatus
